# Exploring animal food microbiomes and resistomes via 16S rRNA gene amplicon sequencing and shotgun metagenomics

**DOI:** 10.1128/aem.02230-24

**Published:** 2025-01-22

**Authors:** Beilei Ge, Ryan C. McDonald, Qianru Yang, Kelly J. Domesle, Saul Sarria, Xin Li, Chih-Hao Hsu, Karen G. Jarvis, Daniel A. Tadesse

**Affiliations:** 1 Office of Applied Science, Center for Veterinary Medicine, U.S. Food and Drug Administration 4137 , Laurel, Maryland, USA; 2 Office of Applied Microbiology and Technology, U.S. Food and Drug Administration 4137 , Laurel, Maryland, USA; 3 Office of Surveillance and Compliance, Center for Veterinary Medicine, U.S. Food and Drug Administration 4137 , Silver Spring, Maryland, USA; INRS Armand-Frappier Sante Biotechnologie Research Centre, Laval, Canada

**Keywords:** 16S rRNA gene amplicon sequencing, animal food, DNA extraction, microbiome, mock community, peptide nucleic acid, resistome, shotgun metagenomics

## Abstract

As a diverse and complex food matrix, the animal food microbiota and repertoire of antimicrobial resistance (AMR) genes remain to be better understood. In this study, 16S rRNA gene amplicon sequencing and shotgun metagenomics were applied to three types of animal food samples (cattle feed, dry dog food, and poultry feed). ZymoBIOMICS mock microbial community was used for workflow optimization including DNA extraction kits and bead-beating conditions. The four DNA extraction kits (AllPrep PowerViral DNA/RNA Kit, DNeasy Blood & Tissue Kit, DNeasy PowerSoil Kit, and ZymoBIOMICS DNA Miniprep Kit) were compared in animal food as well as the use of peptide nucleic acid blockers for 16S rRNA gene amplicon sequencing. Distinct microbial community profiles were generated, which varied by animal food type and DNA extraction kit. Employing peptide nucleic acid blockers prior to 16S rRNA gene amplicon sequencing was comparable with post-sequencing *in silico* filtering at removing chloroplast and mitochondrial sequences. There was a good agreement between 16S rRNA gene amplicon sequencing and shotgun metagenomics on community profiles in animal feed data sets; however, they differed in taxonomic resolution, with the latter superior at resolving at the species level. Although the overall prevalence of AMR genes was low, resistome analysis of animal feed data sets by shotgun metagenomics revealed 10 AMR gene/protein families, including beta-lactamases, erythromycin/lincomycin/pristinamycin/tylosin, fosfomycin, phenicol, and quinolone. Future expansion of microbiome and resistome profiling in animal food will help better understand the bacterial and AMR gene diversity in these commodities and help guide pathogen control and AMR prevention efforts.

**IMPORTANCE:**

With the growing interest and application of metagenomics in understanding the structure/composition and function of diverse microbial communities along the One Health continuum, this study represents one of the first attempts to employ these advanced sequencing technologies to characterize the microbiota and AMR genes in animal food. We unraveled the effects of DNA extraction kits on sample analysis by 16S rRNA gene amplicon sequencing and showed similar efficacies of two strategies at removing chloroplast and mitochondrial reads. The in-depth analysis using shotgun metagenomics shed light on the community compositions and the presence of an array of AMR genes in animal food. This exploration of microbiomes and resistomes in representative animal food samples by both sequencing approaches laid important groundwork for future metagenomic investigations to gain a better understanding of the baseline/core microbiomes and associated AMR functions in these diverse commodities and help guide pathogen control and AMR prevention efforts.

## INTRODUCTION

The United States is one of the major animal food producers in the world, with 238.1 million metric tons produced in 2023 (1) and over $267.1 billion contributed to the U.S. economy (2). Encompassing pet food, animal feed, and raw materials and ingredients (3), this diverse and complex food matrix can be further divided into feed materials/ingredients, feed additives, complete feed (including pet food), and medicated feed (4, 5). A wide range of raw materials and ingredients are used to manufacture animal food, including plant-based materials (e.g., grains and oilseed meals), animal-based materials (e.g., fish meals and meat and bone meals), and feedstuffs of other origins (e.g., vitamins, minerals, amino acids, and stabilizers) (6, 7). In 2023, the global animal food production by sector was as follows: broiler 28.9%, pig 25.2%, layer 13.3%, dairy 10.0%, beef 9.5%, aquaculture 4.2%, pet 2.7%, equine 0.6%, and others 5.6%, with predominant growths in the boiler feed and pet food sectors (1).

Animal food is prone to microbial contamination and may harbor zoonotic pathogens such as *Salmonella enterica* (8) and commensals such as *Escherichia coli* and *Enterococcus* spp. (9). Efforts to isolate and identify other foodborne pathogens or commensals in animal food have only had limited success (10). Although at low frequencies, antimicrobial resistance (AMR) has been observed among pathogenic and commensal bacteria recovered from animal food (8–10). Nonetheless, the comprehensive microbiota (microbiomes) and repertoire of AMR genes (resistomes) in animal food remain poorly characterized and primarily rely on culture-dependent methods that can fail to reveal the true genetic diversity of the community.

Recent years have seen significant technological advancements and cost reductions in the field of next-generation sequencing (11). As such, metagenomic sequencing has been used extensively to profile diverse microbial communities associated with samples from humans, animals, foods, and the environment (12–24). The most common approaches for microbiome characterization are targeted amplicon sequencing of select markers, such as the 16S rRNA gene, and whole metagenome shotgun sequencing of the entire community *en masse* (18). 16S rRNA gene amplicon sequencing provides an affordable means to generate genus-level microbial community profiles, but primer bias and chimera formation may be introduced (25–27). It also provides no insight into the functional capacity of the microbial community. Conversely, shotgun metagenomics can identify various genetic determinants associated with functionality (e.g., AMR genes and virulence factors) with high resolution, but taxonomic classification of all sequencing reads may be challenging as available reference databases still need much improvement (26, 28).

Despite the growing interest and application of metagenomics in understanding the structure/composition and function of diverse microbial communities along the One Health continuum, there is a scarcity of studies using these advanced sequencing technologies to characterize the microbiomes and resistomes in animal food. Similar to human food, animal food constitutes challenging matrices for metagenomic analysis due to variable microbial loads of pathogens and commensals, physicochemical properties that may inhibit DNA extraction and amplification, and high proportions of matrix DNA from plant and animal materials (27, 29). When developing metagenomic workflows for animal food, consideration should be given to core methodologies such as sample preparation, DNA extraction, 16S rRNA gene target regions and amplification protocols, library preparation, multiplexing strategies, bioinformatic tools and algorithms, and reference databases (23, 28).

This study aimed to gain insights into the microbial community and AMR gene profiles of three types of animal food (cattle feed, dry dog food, and poultry feed) by culture-independent 16S rRNA gene amplicon sequencing and shotgun metagenomics. We first used the ZymoBIOMICS mock microbial community (Zymo Research, Irvin, CA) for initial workflow optimization (supplemental material). This optimized workflow was then used to perform two trials in the three types of animal food using entirely different sample sets with replicates. In trial 1, we evaluated the effect of DNA extraction kit and two strategies for removing chloroplast and mitochondria read from the 16S rRNA gene amplicon sequencing data set. In trial 2, we profiled the animal food microbiomes by both 16S rRNA gene amplicon sequencing and shotgun metagenomics and examined resistomes derived from the latter. We present here our exploratory work profiling animal food microbiomes and resistomes using both sequencing approaches.

## MATERIALS AND METHODS

### Animal food samples

Two trials were performed using different sets of bulk animal food samples (18–23 kg) obtained from a local animal food store. These included cattle feed (general-purpose ration for growing and mature beef cattle), dry dog food (complete dog food for all life stages), and poultry feed (complete, general-purpose poultry maintenance feed). From each bulk product, ten 1 kg subsamples were randomly collected and stored at 4°C until analysis.

On the day of analysis, a 100 g composite sample (400 g for dry dog food due to low genomic DNA yield) was formed by combining equal amounts from the 10 subsamples. In trial 1, triplicate 25 g test portions (100 g for dry dog food) of the 100 g composites were aseptically weighed out in Whirl-Pak bags and suspended at a 1:9 ratio in modified buffered peptone water (3M Food Safety, St. Paul, MN). The mixtures were hand-massaged for 5 min (dry dog food was homogenized in a stomacher [Seward, West Sussex, UK] at 230 rpm for 2 min). Four sets (one for each DNA extraction kit) of 25 mL rinsates (210 mL for dry dog food) were transferred to 50 mL Falcon tubes and centrifuged at 900 × *g* for 3 min to remove animal food particles. The supernatants were transferred to new tubes and centrifuged at 10,000 × *g* for 20 min at 8°C. The resulting pellets (12 per sample type; 36 total) were stored at −20°C for DNA extraction by four kits. In trial 2, duplicate 25 g test portions were analyzed by two analysts independently, resulting in a total of 12 pellets for DNA extraction by one kit.

The composite samples were also tested for total aerobic plate counts (APC) by the standard pour plate method (30) and screened for the presence of *Salmonella* according to the U.S. Food and Drug Administration’s *Bacteriological Analytical Manual* (BAM) Chapter 5 (31).

### DNA extraction

In trial 1, four kits, consisting of three from Qiagen (Germantown, MD), namely AllPrep PowerViral DNA/RNA Kit (AllPrep kit in short), DNeasy Blood & Tissue Kit (BloodTissue kit in short), and DNeasy PowerSoil Kit (PowerSoil kit in short), and one from Zymo Research (Irvine, CA), ZymoBIOMICS DNA Miniprep Kit (Zymo kit in short), were used. Three of these kits were bead-based, whereas the BloodTissue kit was enzyme-based. All DNA extraction protocols were performed in triplicate following the manufacturers’ instructions (Gram-positive protocol for the BloodTissue kit) with slight modifications as noted below. In trial 2, DNA extraction was done with the Zymo kit by two analysts independently.

All pellets were pretreated with 20 µL of proteinase K (20 mg/mL, Zymo Research) at 56°C for 1 h before proceeding with DNA extractions, except the BloodTissue kit where proteinase K was part of the Gram-positive protocol. For the Zymo kit, bead-beating used Vortex-Genie 2 for 20 min. The sample DNA extracts were quantified using the Quant-iT Broad-Range or High-Sensitivity dsDNA Assay Kit on a Qubit fluorometer (Thermo Fisher Scientific, Waltham, MA).

### 16S rRNA gene amplicon sequencing

In trial 1, 16S rRNA gene amplicon sequencing was performed through the ZymoBIOMICS Targeted Sequencing Service (Zymo Research), whereas in trial 2, both Zymo service and in-house sequencing were performed. For the Zymo service, all reagents were from Zymo Research unless specified otherwise. Custom primers (proprietary) were used to amplify the 16S rRNA gene V3-V4 region. PCR reactions were performed in the CFX96 Real-Time PCR Detection System (Bio-Rad, Hercules, CA). For animal food, peptide nucleic acid (PNA) blockers were added to prevent the amplification of chloroplast and mitochondrial DNA (32, 33). Sequencing libraries were prepared with the Quick-16S NGS Library Prep Kit. The pooled library was cleaned up with Select-a-Size DNA Clean & Concentrator and quantified with TapeStation (Agilent, Santa Clara, CA) and Qubit. The final 16S library was sequenced on a MiSeq system (Illumina, San Diego, CA) using the MiSeq Reagent Kit v3 (600 cycles) with a 25% PhiX spike-in.

For in-house sequencing, the V3–V4 region of the 16S rRNA gene was targeted with primers Bakt_341F (5′-CCTACGGGNGGCWGCAG-3′) and Bakt_805R (5′-GACTACHVGGGTATCTAATCC-3′) (34). PCR reactions were carried out in a 25 µL volume containing 1× KAPA HiFi HotStart ReadyMix (Roche, Indianapolis, IN), 0.2 µM of each primer, and 2.5 µL of DNA extracts using conditions described previously (34). After purification with AMPure XP (Becker Coulter, Indianapolis, IN) and size verification using TapeStation (Agilent), PCRs were performed to attach dual indices and sequencing adapters using the Nextera XT Index kit (Illumina). Up to 96 libraries (4 nM each) were pooled and sequenced with a 25% PhiX spike-in on MiSeq using the MiSeq Reagent kit v3 with 600 cycles (Illumina).

### Shotgun metagenomics

In trial 2, shotgun metagenomic sequencing libraries of the same DNA extracts used for the in-house 16S rRNA gene amplicon sequencing were constructed using the Nextera XT DNA Library Preparation Kit (Illumina). Briefly, tagmentation and tagging of sample DNA extracts with unique adapter sequences were performed using the Nextera XT transposome. Limited-cycle PCRs were used to amplify the tagged DNA and simultaneously add indexes. After purification with AMPure XP (Becker Coulter), each library was normalized to 4 nM concentration, and equal volumes of normalized libraries were pooled, denatured, and loaded with a final pooled library concentration of 1.8 pM to NextSeq 500 (Illumina) for sequencing using the 500/550 High Output Reagent Kit v2 (300 cycles) (Illumina).

### Taxonomic profiling from the 16S rRNA gene amplicon sequencing data set

QIIME2 (v2023.2) (35) was used for the 16S rRNA gene amplicon sequencing analysis. Briefly, primer sequences and any preceding bases were trimmed from the raw reads using the trim-paired command of the cutadapt plugin (36) with default parameters (--p-error-rate = 0.1). Primer-free reads were error-corrected, and amplicon sequence variants (ASVs) were determined using the denoised-paired command of the DADA2 plugin (37) with default parameters (--p-max-ee-f/r = 2, --p-trunq-q = 2, --p-min-overlap = 12) for quality trimming, read-pair merging, and chimeric sequence removal. Manual forward and reverse read truncation and trimming values were determined based on the average read base scores. For taxonomic classification, the V3–V4 region was extracted from the Silva 138 SSURef NR99 reference database using the primer sequences (--p-min-length = 100, --p-max-length = 700, --p-identity = 0.8) prior to training the naive Bayes classifier. This trained classifier was used to assign taxonomies to ASVs using default parameters.

### Taxonomic and AMR gene profiling of the shotgun metagenomic sequencing data set

Kraken 2 (v 2.1.3) (38) was used for taxonomic profiling with the default k-mer size and parameters. Briefly, base calls generated by the NextSeq 500 System were converted to FASTQ files and trimmed for sequencing adaptors and low-quality sequences using Trimmomatic (39) with parameters (ILLUMINACLIP:Illumina-Adapter.fa:2:30:10 LEADING:20 TRAILING:20 SLIDINGWINDOW:5:20 MINLEN:90). Trimmed and filtered reads were used for all further downstream analyses using the Prebuilt Kraken 2 standard plusPF database (June 2024 update; https://benlangmead.github.io/aws-indexes/k2), which included RefSeq archaea, bacteria, viruses, plasmids, protozoa, fungi, UniVec Core, and the most recent human reference genome (GRCh38). The microbiome composition and the taxa relative abundances were estimated by Bracken (version 2.7) (40) with a default threshold of 10.

AMR gene profiling was performed using the Short, Better Representative Extract Dataset (ShortBRED) (version 0.9.4) (41). First, ShortBRED-Identify was used to generate unique peptide markers for AMR protein sequences compiled from AMRFinderPlus v4.0.1 (database version, 2024–10-29; https://ftp.ncbi.nlm.nih.gov/pathogen/Antimicrobial_resistance/AMRFinderPlus/database/). Specifically, ShortBRED-identify used an 85% amino acid identity threshold to cluster the AMR protein sequences into nonredundant highly conserved protein families. To maintain high specificity, the set of peptides was then blasted against the universal protein reference database UNIREF100 (https://www.uniprot.org/uniref/). ShortBRED-Quantify was used to map translated final sequences at ≥85% amino acid identity across ≥95% of the marker length, normalized to reads per kilobase per million mapped reads (RPKM). Total mapped reads of less than 20 were not considered in the final summary.

### Statistical analysis

All taxonomic and read count data were imported into RStudio (version 2023.12.1) (42) for analysis. Unless stated otherwise, all 16S data sets were rarefied (2,500 minimum read count) prior to calculating alpha and beta diversity measures. Basic alpha diversity measures (observed genera, Simpson’s diversity index, and Pielou’s evenness) were calculated using the vegan community ecology R package (version 2.6–4) (43). Pairwise comparisons between group means were performed using the Wilcox rank-sum test. For beta diversity measurement, Bray-Curtis dissimilarity values were calculated for each pair of samples using vegan. These values were used to perform a principal coordinate analysis (PCoA) using the ecodist package (version 2.0.9). To determine if there are any statistical differences in the community profiles across sample treatment groups, pairwise PERMANOVA was performed using the pairwiseADONIS package (version 0.4.1) (similarity function = “vegdist,” similarity method = “bray,” *P* adjustment method = “holm,” permutations = 9,999). The indicspecies package was used to detect the associations between species patterns and combinations of treatment groups using default parameters (permutations = 999). Mean relative abundances of select taxonomic groups were compared across treatments using a Welch’s *t*-test. Pairwise comparisons between group means were performed using the Wilcox rank-sum test (ANCOM analysis). All plots were visualized using the ggplot2 package (version 3.4.2).

## RESULTS

Benchmarking against a well-defined mock microbial community, we first compared the taxonomic classification using DNA extracted from four commercial kits and analyzed by both 16S rRNA gene amplicon sequencing and shotgun metagenomic sequencing (supplemental material). In animal food trial 1, we evaluated the effects of DNA extraction kit on microbiome analysis and investigated whether applying PNA blockers during 16S rRNA gene amplicon library preparation would effectively inhibit chloroplast and mitochondrial DNA amplification compared with post-sequencing *in silico* filtering of relevant reads from the 16S rRNA gene amplicon sequencing data set. In animal food trial 2, we profiled the microbiomes from both sequencing approaches and resistomes from the shotgun metagenomics data set.

### Workflow optimization with the mock microbial community

Out of the four DNA extraction kits, both metagenomic sequencing approaches showed that the Zymo kit generated taxonomic profiles most closely resembling the mock community composition (Fig. S1 and S2). For this kit, among the nine bead-beating conditions evaluated (PowerLyzer Homogenizer [Qiagen] at 4,000 rpm for 1, 3, and 5 min and at 5,000 rpm for 1 and 3 min, and Vortex-Genie 2 [Scientific Industries, Inc., Bohemia, NY] at maximum speed for 10, 20, 30, and 40 min), bead-beating on Vortex-Genie 2 for 20 min performed best (Fig. S1).

### Animal food microbial counts and DNA extract concentrations by kit

Among the three types of animal food samples, the APCs ranged from 7.9 × 10^2^ CFU/g in dry dog food to 8.7 × 10^2^ CFU/g in poultry feed and to 6.8 × 10^3^ CFU/g in cattle feed.

The AllPrep and Zymo kits yielded the highest average DNA concentrations across all animal food types (3.8 ± 3.9 ng/µL and 2.8 ± 2.7 ng/µL, respectively) with lower yields from the BloodTissue (2.1 ± 0.8 ng/µL) and PowerSoil (2.1 ± 1.8 ng/µL) kits. These differences were not significant as shown in post-hoc comparisons using Tukey’s HSD (all adjusted *P*-values > 0.05). One-way ANOVA revealed that DNA yields varied significantly by animal food type (F (2, 33) = [19.45], *P* = 2.6 × 10^−6^) with cattle feed having the highest DNA concentrations (5.0 ± 3.0 ng/µL), followed by poultry feed (2.7 ± 0.7 ng/µL) and dry dog food (0.4 ± 0.5 ng/µL). Tukey’s HSD also found significant differences in mean DNA concentration among animal food types (all adjusted *P*-values < 0.05) with the highest DNA concentration in cattle feed with the AllPrep kit and the lowest one in dry dog food with the Zymo kit. For dry dog food, which had the lowest overall DNA concentration, the yield was highest using the BloodTissue kit (1.3 ± 0.2 ng/µL), followed by the AllPrep kit (0.4 ± 0.03 ng/µL).

### Animal food microbiomes differed by PNA blocker and DNA extraction kit when analyzed by 16S rRNA gene amplicon sequencing

**Fig 1:**
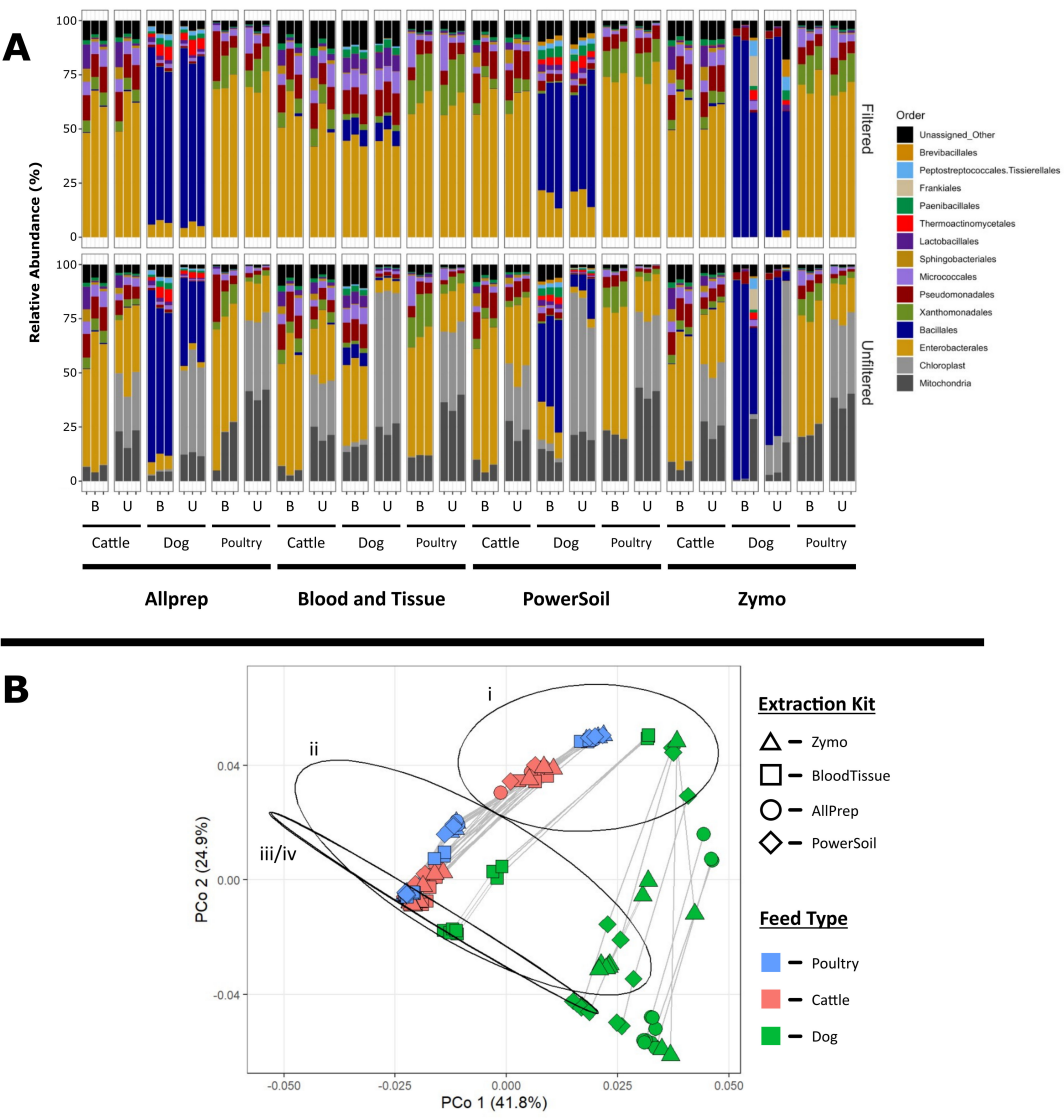
(**A**) Relative abundance of bacterial orders identified using 16S rRNA gene amplicon sequencing in animal food samples processed with different DNA extraction kits in trail 1. Samples were classified as “unblocked” (U) or “blocked” (B) based on the addition of PNA to the PCR reaction mixture and “unfiltered” or “filtered” based on whether microbiomes were depleted of chloroplast and mitochondrial sequence reads bioinformatically. (**B**) PCoA based on Bray-Curtis distances for the animal food samples processed using four DNA extraction kits and analyzed by 16S rRNA gene amplicon sequencing. Samples were grouped based on their blocker and filtering status. Ellipses depict 95% confidence intervals for the unblocked and unfiltered (i), blocked and unfiltered (ii), blocked and filtered (iii), and unblocked and filtered (iv) samples. Gray lines connect samples originating from the same DNA extracts.

In trial 1, the average number of raw reads by 16S rRNA gene amplicon sequencing was 3.3 × 10^4^ ± 1.6 × 10^4^, including large quantities of chloroplast (up to 74.3%) and mitochondrial reads (up to 43.1%). PNA blockers were then applied during 16S rRNA gene amplicon library preparation and compared with post-sequencing *in silico* filtering of chloroplast- and mitochondria-related reads. Distinct microbial communities were observed across the animal food types as well as between blocked and unblocked paired samples (Fig. 1). After *in silico* removal of chloroplast and mitochondrial sequences, cattle and poultry feed communities were dominated by members of the order Enterobacterales from several genera including *Pantoea* (19.8%–31.9%) and *Kosakonia* (3.3%–13.0%) with minor contributions from *Erwinia* and *Klebsiella* (≤ 2%). Both cattle and poultry feed communities also contained elevated levels of orders Pseudomonadales (5.4%–23.0%), Xanthomonadales (3.7%–22.6%), and Micrococcales (1.1%–16.8%). Dry dog food samples possessed a distinct microbial community dominated by Bacillales (6.3%–92.5%) comprised predominantly of the genus *Bacillus* (10.8%–41.9%), with lesser representations by the genera *Virgibacillus, Oceanobacillus*, *Paenibacillus*, and *Pseudogracilibacillus* (Fig. 1A).

Community compositions within dry dog food samples were also highly variable and dependent on the DNA extraction kit (Fig. 1A). The largest differences were observed with the BloodTissue kit where the relative abundance of Bacillales was significantly lower compared with those extracted with other kits (*P* < 0.05). This contrasted with both the cattle and poultry feed samples that showed more consistent community profiles across DNA extraction kits (Fig. 1A).

**Fig 2:**
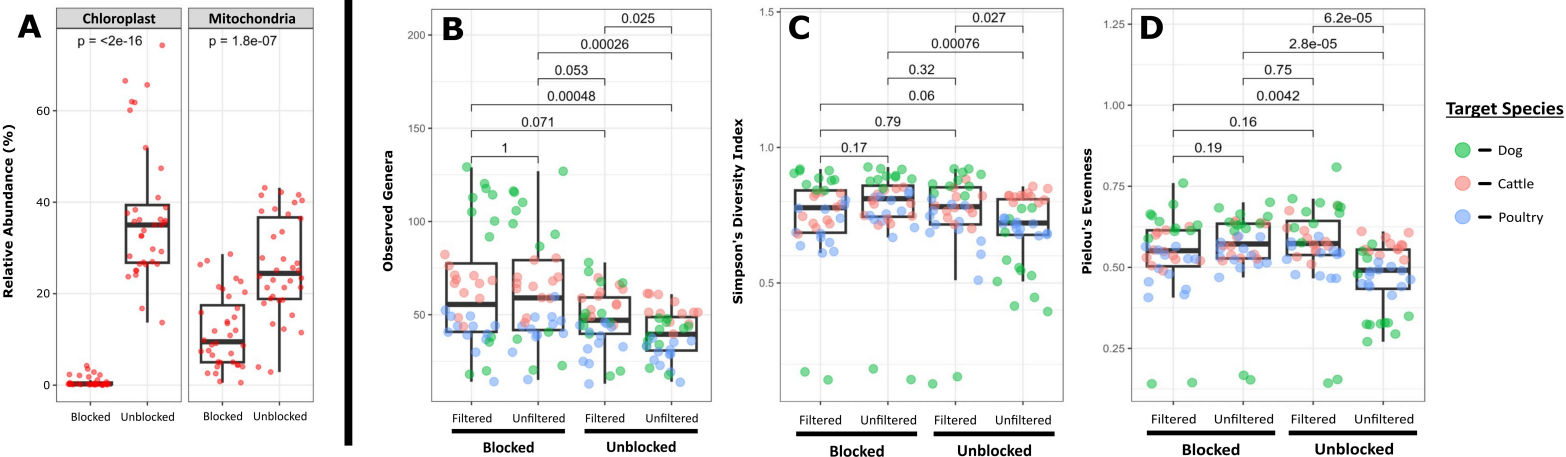
Box-and-whisker plots of (A) chloroplast and mitochondrial relative abundances, (B) Observed genera counts, (C) Simpson’s diversity indices, and (D) Pielou’s evenness measures for animal food microbial communities with and without PNA blockers during library preparation or with and without filtering of 16S rRNA gene amplicon sequencing data sets. Pairwise comparisons between group means were performed using the Wilcox rank-sum test (ANCOM analysis).

Filtering non-bacterial chloroplast and mitochondrial reads from the samples resulted in a significant reduction in the mean number of usable reads between unfiltered and filtered data sets (unfiltered, mean = 4.1 × 10^4^ ± 1.4 × 10^4^ reads; filtered, mean = 2.5 × 10^4^ ± 1.4 × 10^4^ reads; *P* = 5.2 × 10^−10^). In the absence of PNA blockers, the mean fraction of classified microbial reads across all animal food samples was 33.5% ± 18.2%. The addition of PNA blockers prior to sequencing significantly reduced the proportion of chloroplast and mitochondrial sequences even in the absence of filtering (Fig. 2A). However, the blockers were more effective at depleting chloroplast-derived template with a mean reduction of ~99%, whereas there was 57% ± 26% reduction in mitochondrial sequences. Pairwise PERMANOVA performed for each combination of blocked and filtered data showed that both blocking and filtering have a significant impact on overall community composition (Table 1). The only combination of data sets not shown to be significantly different were the two filtered communities (*P* = 0.99) (Table 1; Fig. 2B). Differences in community composition appear to be driven, in part, by the increased biodiversity detected in blocked samples. Compared with other combinations, unblocked and unfiltered samples had significantly lower observed genera counts, as well as Simpson’s diversity and Pielou’s evenness values (Fig. 2B through D). The increased biodiversity detected in blocked samples was further supported by an indicator species analysis that showed 35 genera were significantly associated with blocked samples (Table 2). Those included several genera known to contain important human and animal pathogens such as *Acinetobacter, Clostridium*, *Escherichia*/*Shigella*, and *Peptostreptococcus*. No bacterial genera were significantly associated with unblocked samples suggesting that the addition of blockers did not result in the depletion of microbial sequences.

**TABLE 1:** Pairwise PERMANOVA for animal food communities that were blocked and/or filtered by 16S rRNA gene amplicon sequencing in trial 1

First pair	Second pair	SS*^a^*	F model*^b^*	R^2^^*c*^	Adjusted *P*-value^*d*^
Unblocked and unfiltered	Blocked and unfiltered	4.30	34.5	0.330	0.0006
Unblocked and unfiltered	Unblocked and filtered	6.84	52.1	0.427	0.0006
Unblocked and unfiltered	Blocked and filtered	6.85	52.7	0.430	0.0006
Blocked and unfiltered	Unblocked and filtered	0.66	3.90	0.053	0.0387
Blocked and unfiltered	Blocked and filtered	0.66	3.94	0.054	0.0387
Unblocked and filtered	Blocked and filtered	0.019	0.108	0.002	0.9901

^*a*^ SS stands for the sum of squares.

^*b*^ F model is pseudo-F statistic.

^*c*^ R^2^ is effect size.

^*d*^ *P*-values were adjusted using the Holm–Bonferroni method.

**TABLE 2:** Increased biodiversity in blocked samples exemplified by bacterial genera significantly (*P* < 0.05) associated with those samples by 16S rRNA gene amplicon sequencing in trial 1

Phylum	Class	Order	Family	Genus	*P*-value
Actinomycetota	Actinobacteria	Kineosporiales	Dermabacteraceae	*Brachybacterium*	0.001
			Microbacteriaceae	*Microbacterium*	0.002
				*Schumannella*	0.02
			Kineosporiaceae	*Kineococcus*	0.003
			Promicromonosporaceae	*Cellulosimicrobium*	0.005
Firmicutes	Bacilli	Bacillales	Bacillaceae	*Geobacillus*	0.031
				*Ureibacillus*	0.015
			Planococcaceae	*Kurthia*	0.018
				*Lysinibacillus*	0.001
				*Rummeliibacillus*	0.01
				*Sporosarcina*	0.009
		Lactobacillales	Aerococcaceae	Unclassified	0.031
				*Aerococcus*	0.015
		Paenibacillales	Paenibacillaceae	Uncultured	0.023
		Thermoactinomycetales	Thermoactinomycetaceae	*Melghirimyces*	0.035
				*Planifilum*	0.031
				Uncultured	0.009
	Clostridia	Clostridiales	Clostridiaceae	*Clostridium_sensu_stricto_15*	0.009
				*Hathewaya*	0.017
		Lachnospirales	Lachnospiraceae	*Anaerosporobacter*	0.02
		Peptostreptococcales.Tissierellales	Peptostreptococcaceae	*Peptostreptococcus*	0.005
				Unclassified	0.017
				*Peptoniphilus*	0.001
Proteobacteria	Alphaproteobacteria	Acetobacterales	Acetobacteraceae	*Acetobacter*	0.001
		Rhizobiales	Rhizobiaceae	Uncultured	0.016
			Xanthobacteraceae	*Bradyrhizobium*	0.003
	Gammaproteobacteria	Aeromonadales	Aeromonadaceae	*Aeromonas*	0.025
		Burkholderiales	Alcaligenaceae	*Advenella*	0.032
				*Alcaligenes*	0.002
			Comamonadaceae	*Xylophilus*	0.032
			Oxalobacteraceae	*Janthinobacterium*	0.008
		Enterobacterales	Enterobacteriaceae	*Escherichia.Shigella*	0.001
			Yersiniaceae	*Rahnella1*	0.004
				Unclassified	0.032
		Xanthomonadales	Xanthomonadaceae	*Luteimonas*	0.011

### Animal food microbiome comparisons by 16S rRNA gene amplicon sequencing and shotgun metagenomics side-by-side

In trial 2, an average of 172,308 (range: 128,247–227,580) and 15,314,408 (range: 11,692,126–24,541,655) reads were obtained across all animal food samples by 16S rRNA gene amplicon sequencing and shotgun metagenomics, respectively. Species accumulation curves were generated for all 16S rRNA gene amplicon sequencing and shotgun metagenomics samples (data not shown). All curves reached stationarity, suggesting that increased sampling effort would not significantly increase the number of observed microbial species. An average of 42.0% and 6.1% reads were mapped to bacteria by 16S rRNA gene amplicon sequencing and shotgun metagenomics, respectively. Dry dog food was not able to be analyzed by shotgun metagenomics due to low DNA concentrations (< 0.2 ng/µL) in all replicates.

**Fig 3:**
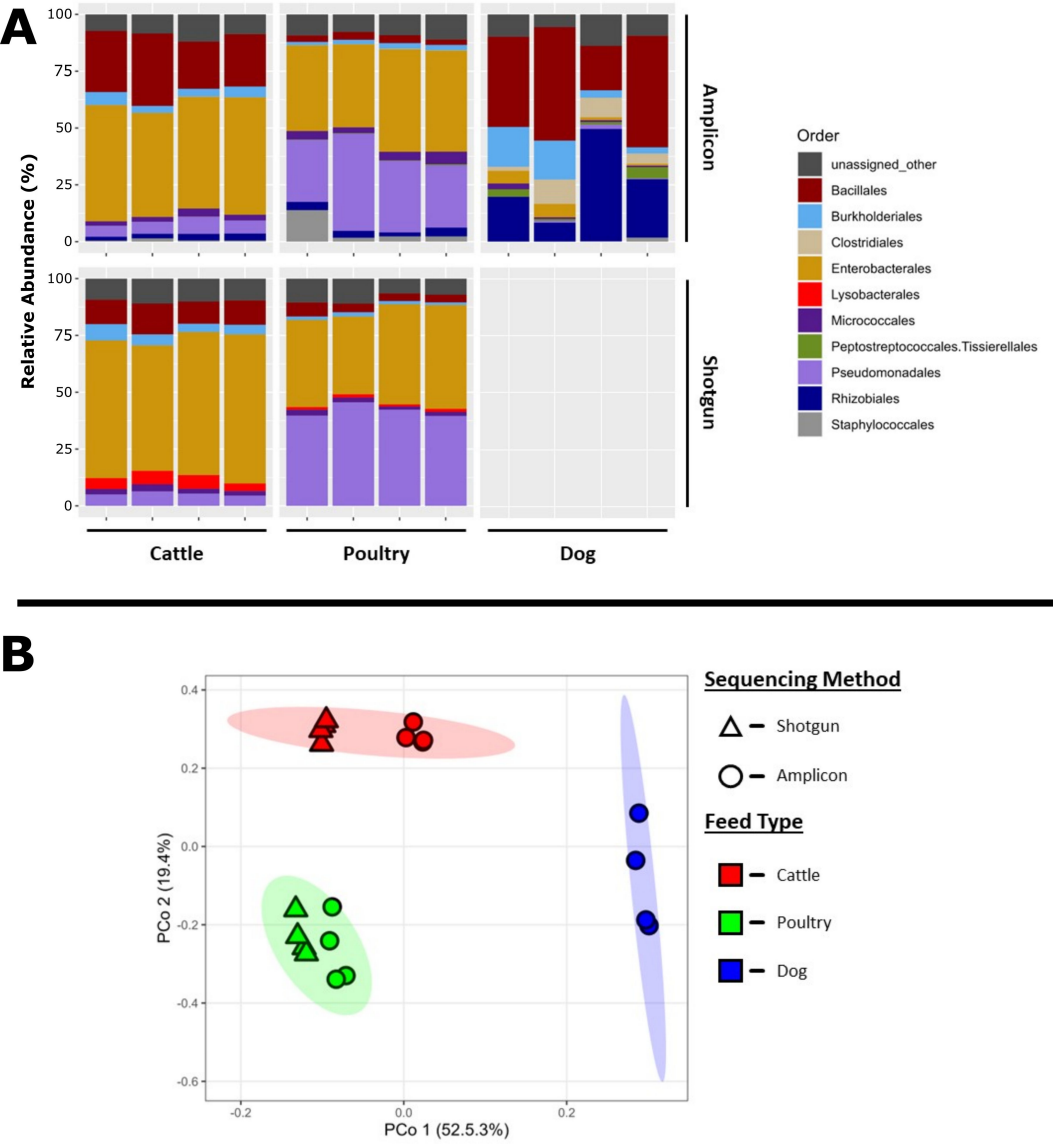
(**A**) Relative abundances of bacterial orders identified using 16S rRNA gene amplicon sequencing and shotgun metagenomics in animal food samples in trial 2. (**B**) PCoA based on Bray-Curtis distances for the animal food samples using the two sequencing approaches. Ellipses depict 95% confidence intervals for color-coded sample types.

Consistent with trial 1, distinct microbial communities were identified across the three animal food types (all *P* < 0.05) (Fig. 3). Both cattle and poultry feed samples were dominated by the members of the orders Bacillales, Enterobacterales, and Pseudomonadales, whereas the dry dog food samples had a more diverse community comprised predominantly of Bacillales, Burkholderiales, Clostridiales, and Rhizobiales. The choice of the sequencing approach did not significantly alter the community profiles within animal food types (all *P* > 0.25); however, differences could still be observed in relative abundances at the order taxonomic rank (Fig. 3A). A limited number of bacterial orders were associated with specific sequencing approaches. These included Rhizobiales and Staphylococcales, which were identified exclusively in samples analyzed by 16S rRNA gene amplicon sequencing, whereas the order Lysobacterales was identified exclusively in samples sequenced using shotgun metagenomics.

Differences in community profiles between the two sequencing approaches were also observed at the genus level within the predominant microbial orders. The primary genera included *Pantoea* (18.6%–39.2%), *Enterobacter* (0%–18.3%), *Kasakonia* (2.9%–13.7%), and *Klebsiella* (0%–5.0%). Detection of *Pantoea, Enterobacter,* and *Kasakonia* appeared to be independent of the sequencing approach, as they were identified at similar frequencies across all samples. However, *Klebsiella* was identified at a higher rate in shotgun metagenomics data set (3.5% ± 0.9%) than 16S rRNA gene amplicon sequencing data set (0.5% ± 0.7%) (*P* = 1.05 × 10^−7^). A similar trend was observed within the order Bacillales, with the primary genera identified as *Bacillus* (cattle and poultry feeds) and *Anaerobacillus* (dry dog food) using the 16S rRNA gene amplicon sequencing. This contrasted with the shotgun metagenomics data set, which contained predominantly *Priestia* for cattle and poultry feeds (no dry dog food shotgun data available).

**Fig 4:**
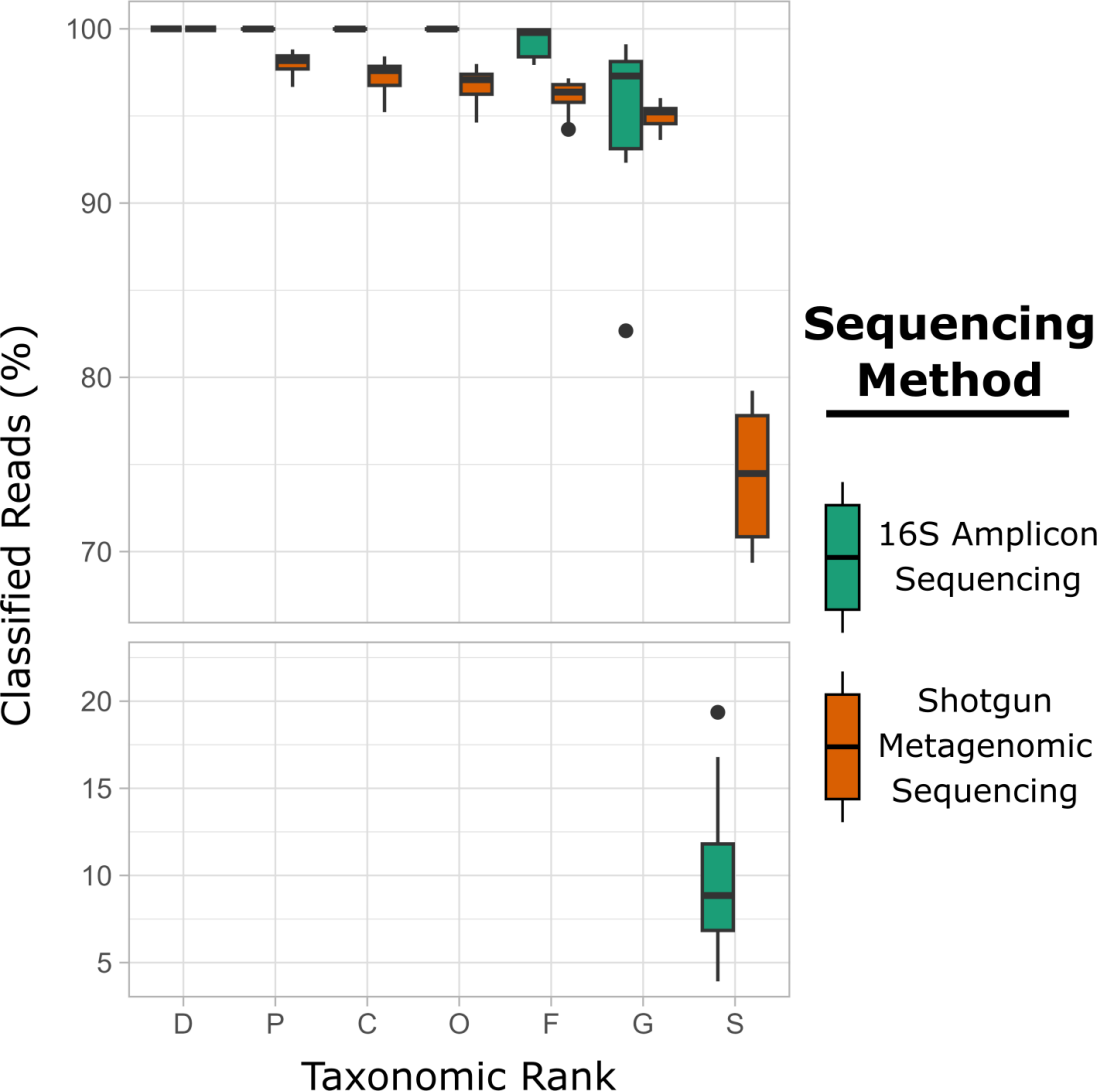
Relative abundance of microbial reads successfully classified at different taxonomic ranks by the two sequencing approaches, 16S rRNA gene amplicon sequencing versus shotgun metagenomics. D – domain, P – phylum, C – class, O – order, F – family, G – genus, and S – species.

Direct species-level comparisons between 16S rRNA gene amplicon and shotgun metagenomic sequences were not possible due to drastic differences in taxonomic resolution (Fig. 4). Generally, both methods were able to successfully classify microbial reads down to the genus level with 16S rRNA gene amplicon sequencing outperforming shotgun metagenomics at higher taxonomic ranks. However, at the species level, shotgun metagenomics analysis was able to classify 74.4% ± 4.1% of microbial reads, which was significantly higher than 16S rRNA gene amplicon sequencing of 9.9% ± 4.8% (*P* < 0.05).

### Animal food resistomes by shotgun metagenomic sequencing

In trial 2, shotgun metagenomic sequencing of cattle and poultry feed samples revealed 10 AMR gene/protein families. The relative abundances of the detected AMR genes are shown in Table 3. Although the overall prevalence of AMR genes in animal feed samples was low, we identified resistance genes encoding beta-lactamases (*bla*_CMH-1_ and *bla*_ACT-GC1_ gene/protein families), erythromycin/lincomysin/pristinamycin/tylosin (*erm*(O)), quinolone (*qnrE2* gene/protein family), and fosfomycin (*fosA8*) from cattle feed. Additionally, we found phenicol/quinolone resistance genes (*oqxA2* and *oqxB29* gene/protein families) in both cattle and poultry feed samples. Furthermore, we identified three multidrug (MDR) efflux pump genes (*emrD*, *norM*, and *kdeA*) that have the potential to confer resistance to different antimicrobials and dyes.

**TABLE 3:** Antimicrobial resistance genes identified in cattle and poultry feed samples by shotgun metagenomics in trial 2

Sample	Relative abundance of antimicrobial resistance genes (RPKM)*^a^*									
	*bla*_CMH-1_ family*^b^*	*bla*_ACT-GC1_ family*^b^*	*emrD*	*erm*(O)	*oqxB29* family^*b*^	*oqxA2* family^*b*^	*qnrE2* family^*b*^	*fosA8*	*norM*	*kdeA*
Cattle feed 1		2.178			3.421					
Cattle feed 2	6.696	1.674			3.348	3.805				
Cattle feed 3		1.439	1.222	1.019		0.926		0.774		3.704
Cattle feed 4	2.410		1.205		4.819		2.552		1.369	
Poultry feed 1										
Poultry feed 2										
Poultry feed 3					2.797					
Poultry feed 4			1.158			1.316				

^*a*^ RPKM stands for reads per kilobase per million mapped reads.

^*b*^ An 85% amino acid identity threshold was used to cluster the AMR gene/protein sequences into nonredundant highly conserved protein families.

## DISCUSSION

This study is one of the first attempts employing both 16S rRNA gene amplicon sequencing and shotgun metagenomics to characterize the microbiomes and resistomes in three types of animal food products. Our analysis workflows include genomic DNA extraction, PCR amplification of the 16S V3–V4 region (for 16S rRNA gene amplicon sequencing only), library preparation, sequencing on MiSeq (for 16S rRNA gene amplicon sequencing) and NextSeq (for shotgun metagenomics), and bioinformatic analysis (read processing, taxonomy classification, and AMR gene identification). Since there are no previous studies focused on metagenomic analyses of animal food, we pivoted our efforts to determine which DNA extraction method(s) most accurately reconstructed the mock community structure. We followed up by evaluating the workflows in animal food and showed the critical need to remove chloroplast and mitochondrial DNA using two different strategies. Finally, we obtained microbiomes in these animal food sample types using both sequencing approaches and resistomes using shotgun metagenomics.

Many surveys have indicated that the bacterial genera/species in the ZymoBIOMICS mock microbial community included those commonly found in animal food such as *Salmonella*, *E. coli*, *Enterococcus*, *Listeria*, *Bacillus*, and *Lactobacillus* (8, 9, 44–46). Nonetheless, we acknowledge a major limitation of this study, which is not spiking the mock community in animal food and analyzing that mixture *en masse*. As shown in the supplemental material, our mock community analysis demonstrated that the AllPrep and Zymo kits were superior among the four kits tested, at extracting genomic DNA from the eight bacteria using 16S rRNA gene amplicon and shotgun metagenomic sequencing. The PowerSoil and Zymo kits were able to extract genomic DNA from the two fungi, shown in the shotgun metagenomics data set. The analyses also indicated that bead-beating on Vortex-Genie 2 for 20 min was optimal for the Zymo kit. These represent important efforts toward standardization of an essential step in both sequencing workflows. Similar efforts to standardize DNA extraction methods have been reported in human gut and urine samples (47, 48). Typically, extended bead-beating times are associated with greater DNA sheering and reduced microbial diversity as they could lead to loss of fragments, incomplete or inefficient amplification, and inaccurate representation of the microbial community diversity. However, consistent community profiles were observed even after 40 min of bead-beating using the Vortex Genie. This is likely due to the lower speed (~3,000 rpm) and relative inefficiency of benchtop vortexers compared with dedicated tissue homogenizers. A recent study compared various lysis protocols (thermal, enzymatic, and mechanical [bead]), and for the bead-based method, over 40 different bead material/size combinations and cell disruptor type/intensity/run time combinations; this study proposed the use of Measurement Integrity Quotient score (range: 0–100, assigned by measuring the root mean square error of observed relative abundances that fall outside the band of manufacturing tolerance, which is 15% for the ZymoBIOMICS mock community) for easy comparison of the methods, in order to reduce bias associated with DNA extraction (49).

When applied to animal food, not surprisingly, taxonomic profiles generated using 16S rRNA gene amplicon sequencing varied greatly based on the DNA extraction kits used (Fig. 1). It is also noteworthy that the concentrations of DNA extracts in animal food samples (average: 0.4–5.0 ng/μL) were lower than those for the mock community (average: 10.6 ng/µL), particularly for dry dog food. Industrial dry dog food is usually processed at temperatures of 80–160°C under high pressure during the extrusion step, which significantly reduces the bacterial load. Since we used a pelleting method to obtain microbial cells from the animal food samples, the lack of intact cells greatly reduced the size of the pellets, thereby resulting in lower DNA concentrations being extracted. A recent study showed that APCs in 50% of the dry dog food samples tested did not exceed 10^2^ CFU/g (50). Nonetheless, the difficulty with extracting sufficient concentrations of genomic DNA from animal food, in general, contributed in part to it being a challenging matrix for metagenomic analysis.

Our preliminary data clearly indicated that chloroplast and mitochondria consumed significant amounts of 16S rRNA gene amplicon sequencing reads (Fig. 1). However, we found that the application of 16S rRNA sequencing PNA blockers prior to PCR amplification significantly reduced these non-bacterial contaminants in the final community profile. The reduction of chloroplast and mitochondrial reads in the sequencing data sets had the added benefit of increasing the detection rate of relatively low abundant microorganisms (Table 2). This is particularly useful in food microbiology studies where pathogenic organisms of interest can exist at concentrations at or below the level of detection using marker gene surveys. If the study design does not require the detection of rare species or depth of sequencing is not a consideration, it may be advisable to forego the application of PNA blockers and simply remove the chloroplast and mitochondrial sequence reads from the data sets post-sequencing. In this study, both blocked and unblocked samples had similar community profiles after filtering across diverse animal food types. Several robust tools have been developed and incorporated into the bioinformatic pipelines used for targeted gene sequence analyses, making the removal of contaminating sequence reads easier.

It is widely accepted that 16S rRNA gene amplicon sequencing and shotgun metagenomic sequencing generally serve two different purposes for microbiome analysis (51). Targeting the 16S rRNA gene allows the identification of relatively high and low abundant taxa and is economical. Shotgun metagenomic sequencing allows for high-resolution taxonomic classification due to the higher sequencing depth and broader genome coverage compared with 16S rRNA gene amplicon sequencing, and it is capable of identifying genomic features such as serotypes, AMR genes, and virulence factors, among others. In our study, both sequencing approaches allowed comprehensive characterization of bacterial diversity in the three types of animal food samples, although the resolution for 16S rRNA gene amplicon sequencing was obviously lower (shown at the species level) (Fig. 4). Interestingly, we observed that 16S rRNA gene amplicon sequencing had better resolutions than shotgun metagenomics at higher taxonomic ranks (phylum to genus; Fig. 4), which corroborated with a recent report (52). Shotgun metagenomic sequencing required higher quality DNA or a better multiplexing strategy, as some samples did not pass the quality filter or read threshold. This limitation was more prominent in resistome characterization. For instance, dry dog food proved to be a challenging matrix and yielded low DNA concentrations. Low microbial biomass and high concentrations of matrix components resulted in the majority of reads (93.9%) in the shotgun metagenomic data set being unclassified. Many of these reads may be attributed to non-prokaryotic DNA or to previously uncultured/uncharacterized microorganisms. A global metagenomic analysis of AMR in sewage reported that across all data sets, only 0.3% of the reads were assigned to 16/18S rRNA, and of these, 96.8% and 2.9% were mapped to bacteria and eukaryotes, respectively, with just 0.05% of the reads assigned to AMR genes (53). All of these factors led to challenges with both taxonomic composition and AMR gene identification in complex animal food communities.

The predominant microbial taxa varied by animal food type using both sequencing approaches (Fig. 1 and 3). The differences in community composition are likely due to different base ingredients used to make the finished product. We identified 10 AMR gene/protein families conferring resistance to multiple antimicrobials (Table 3). These warrant further studies where larger sample sizes are examined. For studies where the only interest is in microbial community composition, both 16S rRNA gene amplicon sequencing and shotgun metagenomics may be used. However, when identifying AMR genes is an essential requirement, shotgun metagenomics should be the technique of choice. Nonetheless, this study provides a snapshot of in-depth microbiomes and resistomes in animal food using both sequencing approaches. These promising next-generation sequencing technologies, upon further standardization, will be valuable tools to help better understand the bacterial and AMR gene diversity in animal food to guide pathogen control and AMR prevention efforts.

## Data Availability

The 16S rRNA gene amplicon sequencing and shotgun metagenomic sequencing data sets for animal food sample DNA extracts were deposited at the National Center for Biotechnology Information (NCBI)’s Sequence Read Archive (SRA) database under BioProject accession numbers PRJNA1093005 and PRJNA1093003, respectively.

## SUPPLEMENTAL MATERIAL

Supplemental material aem.02230-24-s0001.docx Mock community experiments.

ASM does not own the copyrights to Supplemental Material that may be linked to, or accessed through, an article. The authors have granted ASM a non-exclusive, world-wide license to publish the Supplemental Material files. Please contact the corresponding author directly for reuse.
