# Supplementary material for: Exploring animal food microbiomes and resistomes via 16S rRNA gene amplicon sequencing and shotgun metagenomics
Source: Appl Environ Microbiol. 2025 Jan 22;91(2):e02230-24. doi: 10.1128/aem.02230-24 (PMC11837513; doi:10.1128/aem.02230-24)
Supplement: Supplemental material — Mock community experiments. [file aem.02230-24-s0001.docx]

**Supplemental Material:**

**Exploring Animal Food Microbiomes and Resistomes via 16S rRNA Gene Amplicon Sequencing and Shotgun Metagenomics**

Beilei Ge^1*^, Ryan C. McDonald^1^, Qianru Yang^1^, Kelly J. Domesle^1^, Saul Sarria^1^, Xin Li^2^, Chih-Hao Hsu^1^, Karen G. Jarvis^3^, and Daniel A. Tadesse^1^

^1^Office of Applied Science, Center for Veterinary Medicine, U.S. Food and Drug Administration, Laurel, MD 20708, USA

^2^Office of Surveillance and Compliance, Center for Veterinary Medicine, U.S. Food and Drug Administration, Rockville, MD 20855, USA

^3^Office of Applied Microbiology and Technology, U.S. Food and Drug Administration, Laurel, MD 20708, USA

#Current affiliation: Office of Applied Microbiology and Technology, U.S. Food and Drug Administration, Laurel, MD 20708, USA

*Corresponding author. Address: Office of Applied Science, Center for Veterinary Medicine, U.S. Food and Drug Administration, 8401 Muirkirk Road, Laurel, MD 20708. Phone: +1 (240) 402-5452. Fax: +1 (301) 210-4685. Email: [beilei.ge@fda.hhs.gov](mailto:beilei.ge@fda.hhs.gov). ORCID iD: 0000-0001-7995-7427

## **MATERIALS AND METHODS**

**Mock microbial community.** The ZymoBIOMICS Microbial Community Standard (Zymo Research, Irvine, CA) was used for workflow benchmarking. This mock community contained ten microorganisms: 5 Gram-positive bacteria (*Bacillus subtilis*, *Enterococcus faecalis*, *Limosilactobacillus fermentum*, *Listeria monocytogenes*, and *Staphylococcus aureus*), 3 Gram-negative bacteria (*E. coli*, *Pseudomonas aeruginosa*, and *S. enterica*), and 2 fungi (*Cryptococcus neoformans* and *Saccharomyces cerevisiae*) with GC contents ranging from 15-85%. The culture mixture was fully inactivated using DNA/RNA Shield (a DNA/RNA stabilization solution, Zymo Research) and pooled to desired ratios (12% genomic DNA relative abundance for each bacterium and 2% genomic DNA relative abundance for each fungus).

**DNA extraction.** The same four kits (AllPrep, BloodTissue, PowerSoil, and Zymo) used for DNA extraction from animal food samples were used for extraction from the mock microbial community. Briefly, the standard 75-μl volume was used for BloodTissue and Zymo kits, whereas a modified 15-μl volume was used for AllPrep and PowerSoil kits per Zymo Research’s instruction (personal communication). The optimal bead-beating conditions for the Zymo kit were evaluated using the PowerLyzer Homogenizer (Qiagen, Germantown, MD) at 4,000 rpm for 1, 3, and 5 min and at 5,000 rpm for 1 and 3 min, as well as using the Vortex-Genie 2 (Scientific Industries, Inc., Bohemia, NY) at maximum speed for 10, 20, 30, and 40 min. All bead-beating was performed at 22°C.

**Metagenomic sequencing and bioinformatic analysis.** Procedures described in the main text were followed, including sequencing using 16S rRNA gene amplicon sequencing and shotgun metagenomics, taxonomic profiling from both sequencing datasets and antimicrobial resistance gene profiling of the shotgun metagenomics dataset, as well as statistical analysis.

As a reference to the amplicon sequence variants (ASV) analysis, an *in silico* 16S rRNA gene sequence dataset (1×10^6^ reads total) was established. Relative abundance of full-length 16S rRNA gene sequences was determined based on the mock community’s theoretical composition (<https://files.zymoresearch.com/datasheets/ds1706_zymobiomics_microbial_community_standards_data_sheet.pdf>). For each microorganism, reads were evenly distributed across each of the sequence variants among multiple 16S rRNA gene copies (Table 1). Naïve Bayes classifiers were trained for the full-length 16S sequences dataset (1). ASV counts and assigned taxa were recorded and used in downstream analyses including taxonomic characterization, alpha and beta diversity, and principal coordinate analysis (PCoA).

## **RESULTS**

**The choice of DNA extraction kit affected mock community taxonomic profiling by 16S rRNA gene amplicon sequencing.** Table S1 outlines theoretical relative abundances of microorganism composition values and read characteristics of the mock microbial community extracted with the Zymo kit and analyzed by 16S rRNA gene amplicon sequencing.

Fig. S1A shows genus-level taxonomic profiles from 16S rRNA gene amplicon sequencing of the mock community extracted with the AllPrep, BloodTissue, PowerSoil, and Zymo kits. While all eight bacterial genera and species were identified by each kit, extraction efficiencies varied greatly. The BloodTissue and PowerSoil kits resulted in significant lower relative abundances of Gram-positive organisms particularly *Listeria* (98±2% reduction for BloodTissue and 59±2% reduction for PowerSoil), while overestimating Gram-negative organisms *Escherichia*, *Pseudomonas*, and *Salmonella* relative abundances (Fig. S1A).

The finding is supported by PCoA analysis (Fig. S1B). Mock community DNA extracted with the Zymo and AllPrep kits showed the highest agreement with the theoretical community composition based on Bray-Curtis dissimilarity measures, whereas showed a greater and significant divergence (pairwise PERMANOVA (ADONIS), both *p* < 0.01) (Fig. S1B).

**The conditions of bead-beating affected mock community taxonomic profiling when using Zymo kit and analyzed by 16S rRNA gene amplicon sequencing.** While the overall performance of the Zymo extraction kit was superior to the other three kits, it was highly dependent on bead-beating conditions (Fig. S1A). Zymo samples homogenized using the Vortex-Genie 2 showed consistent microbial community profiles that did not vary significantly across the various bead-beating durations (*p* = 0.06). For Zymo samples processed using the PowerLyzer Homogenizer, bead-beating intensity (rpm) significantly impacted the community profiles (*p* < 0.001), while duration did not (*p* = 0.453). The use of the PowerLyzer homogenizer at 5,000 rpm for 3 min resulted in significant reductions in the relative abundances of *Escherichia*, *Pseudomonas*, and *Salmonella*, and overestimations of *Listeria* and *Enterococcus* (all *p* < 0.01). These deviations from the theoretical relative abundances were largely mitigated when the bead-beating duration was reduced to 1 min or the intensity was reduced to 4,000 rpm (Fig. S1A).

The above findings were supported with principal coordinate analysis (PCoA) based on Bray-Curtis dissimilarity metrics (Fig. S1B). 16S rRNA gene samples prepared using the BloodTissue, PowerSoil, and PowerLyzer (at 5,000 rpm)-processed Zymo DNA extractions generated taxonomic profiles furthest from the mock community’s theoretical profile. Samples with Vortex-Genie 2 as the bead-beating apparatus and those using PowerLyzer at 4,000 rpm for the Zymo kit and those extracted by the AllPrep kit had community profiles cluster closely with the theoretical mock community profile. Of these, the Zymo kit with beat-beating on the Vortex-Genie 2 for 20 min had the smallest Bray-Curtis dissimilarity value from the theoretical mock community (Fig. S1B).

**The choice of DNA extraction kit affected mock community taxonomic profiling by shotgun metagenomic sequencing.** Consistent with the 16S rRNA gene amplicon dataset, the choice of DNA extraction method had a major impact on the mock microbial community profiling when analyzed by shotgun metagenomics (Fig. S2A and S2B). The eukaryotic fractions (*Cryptococcus* and *Saccharomyces*) of the mock community, a feature not detectable using 16S rRNA gene amplicon sequencing, were only identified in samples processed by the Zymo kit in (samples homogenized using Vortex-Genie 2 for 20 min) and the PowerSoil kit (Fig. S2A and S2B). For the prokaryotic fractions, similar to the 16S rRNA sequencing, samples processed by the Zymo and AllPrep kits generated comparable taxonomic classifications closely mimicking the mock community compositions. *Pseudomonas* had the highest divergence from the mock community in the shotgun metagenomics dataset with a mean reduction of 67±8% for the Zymo kit and 61±6% for the AllPrep kit (both *p* < 0.01). The BloodTissue kit showed significant reductions (99%) in the relative abundance of *Listeria* (*p* < 0.01), whereas both *Listeria* (64±5%) and *Enterococcus* (61±5%) were underrepresented in samples extracted by the PowerSoil kit (*p* < 0.01) (Fig. S2A).

The PCoA with Bray-Curtis dissimilarity values again indicated the better performance of the AllPrep and Zymo kits in generating taxonomic profiles matching with those of the mock community, based on both metagenomic sequencing approaches (Fig. S2B).

## **DISCUSSION**

Our mock community analysis demonstrated that the AllPrep and Zymo kits were superior among the four kits tested, at extracting DNA from the eight bacterial genomes using 16S rRNA amplicon and shotgun metagenomic sequencing. The PowerSoil and Zymo kits were able to extract genomic DNA from two fungi, shown in the shotgun metagenomic dataset. The analyses also indicated that bead-beating on Vortex-Genie 2 for 20 min was optimal for the Zymo kit. These represent important efforts towards standardization of an essential step in both metagenomic sequencing workflows. It is interesting to note that bead-beating intensity not duration had the most effect on the sequencing outcome when using the Zymo kit. At higher intensity, we had observed tubes broken after a few min, resulting unusable samples. It is critical to pay attention to follow the recommended bead-beating protocols when performing DNA extraction using this kit.

## **REFERENCES**

1. Pruesse E, Quast C, Knittel K, Fuchs BM, Ludwig W, Peplies J, Glockner FO. 2007. SILVA: a comprehensive online resource for quality checked and aligned ribosomal RNA sequence data compatible with ARB. Nucleic Acids Res 35:7188-96.

## **FIGURES**

**Figure S1.** (A) Stacked bar charts depicting the relative abundance of bacterial genera identified in the mock community using the ZymoBIOMICS DNA Miniprep Kit (ZymoBIOMICS), AllPrep PowerViral DNA/RNA Kit (AP), DNeasy Blood & Tissue Kit (BT), and DNeasy PowerSoil Kit (PS) when analyzed by 16S amplicon sequencing. In addition to comparisons between DNA extraction kits, bead-beating conditions were optimized for the ZymoBIOMICS kit and included the comparisons between bead-beating platforms (Vortex-Genie 2 and PowerLyzer), intensities (4,000 and 5,000 rpm), and time intervals. To assess the effectiveness of the kits and bead-beating conditions, community profiles were compared to the published theoretical composition (M). (B) Principal coordinate analysis (PCoA) based on Bray-Curtis dissimilarity for mock microbial communities prepared using the AllPrep (triangle), BloodTissue (square), Zymo (circle), and PowerSoil (diamond) kits. The relative fidelity of the kits was determined based on dissimilarity from the theoretical mock community profile (asterisk).

**A
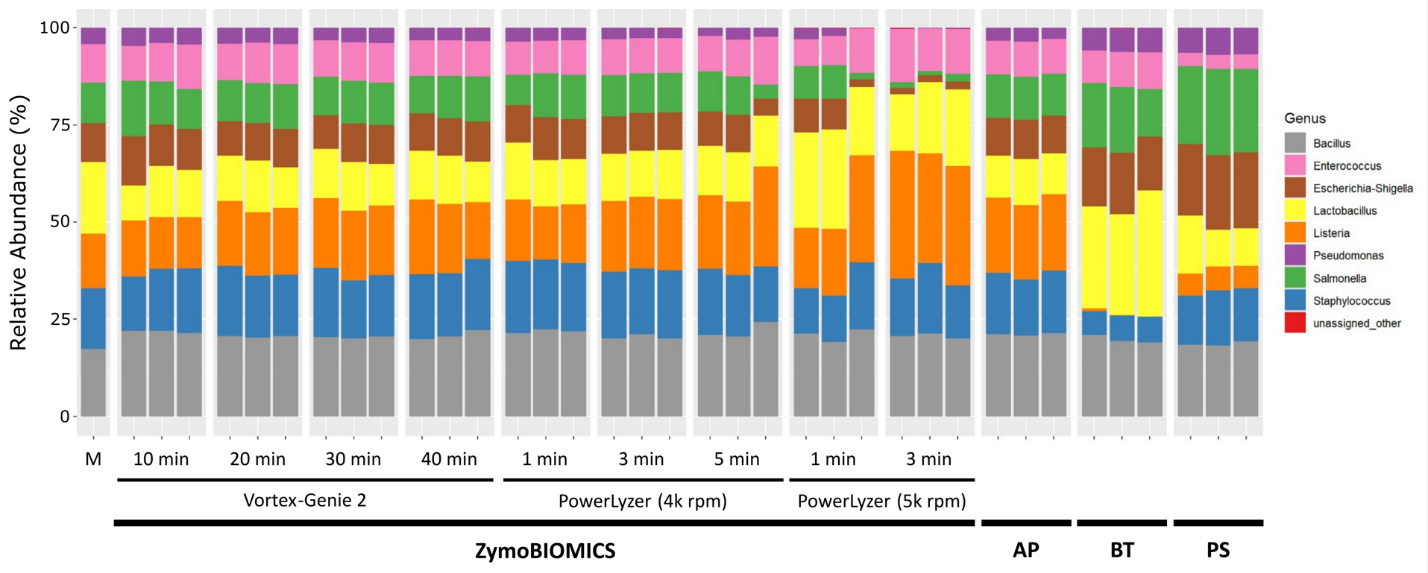
**

**B**

**
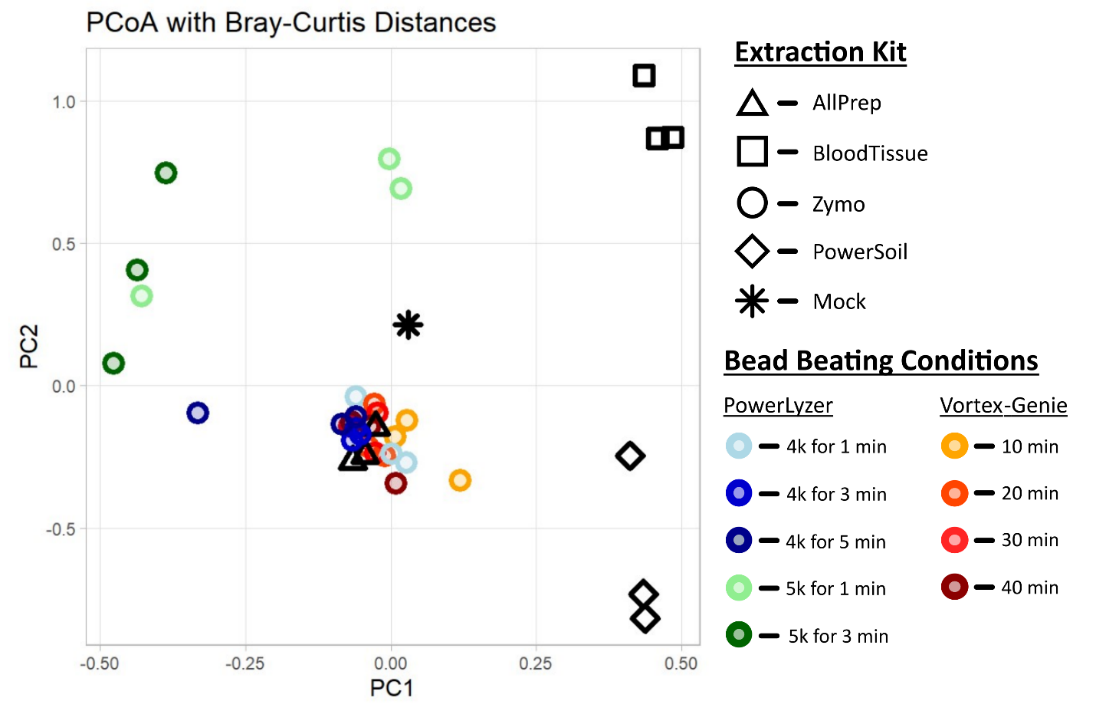
**

**Figure S2**. (A) Stacked bar charts depicting the relative abundance of microbial genera identified in the mock community using the ZymoBIOMICS DNA Miniprep Kit (Zymo), AllPrep PowerViral DNA/RNA Kit (AP), DNeasy Blood & Tissue Kit (BT), DNeasy PowerSoil Kit (PS) analyzed by 16S rRNA gene amplicon sequencing (ASV) and shotgun metagenomic sequencing (relative abundance). The Zymo bead-beating used the Vortex-Genie 2 for 20 min. To assess kit fidelity, community profiles were compared to the published theoretical compositions (M). (B) PCoA based on Bray-Curtis dissimilarity for mock microbial communities prepared using the AllPrep (circle), BloodTissue (square), Zymo (triangle), and PowerSoil (diamond) kits. Dissimilarity values were calculated using taxonomic relative abundance at the genus level. Gray lines connect samples originating from the same DNA extract but were sequenced using using shotgun metagenomic sequencing or 16S rRNA gene amplicon sequencing.

**A**

**
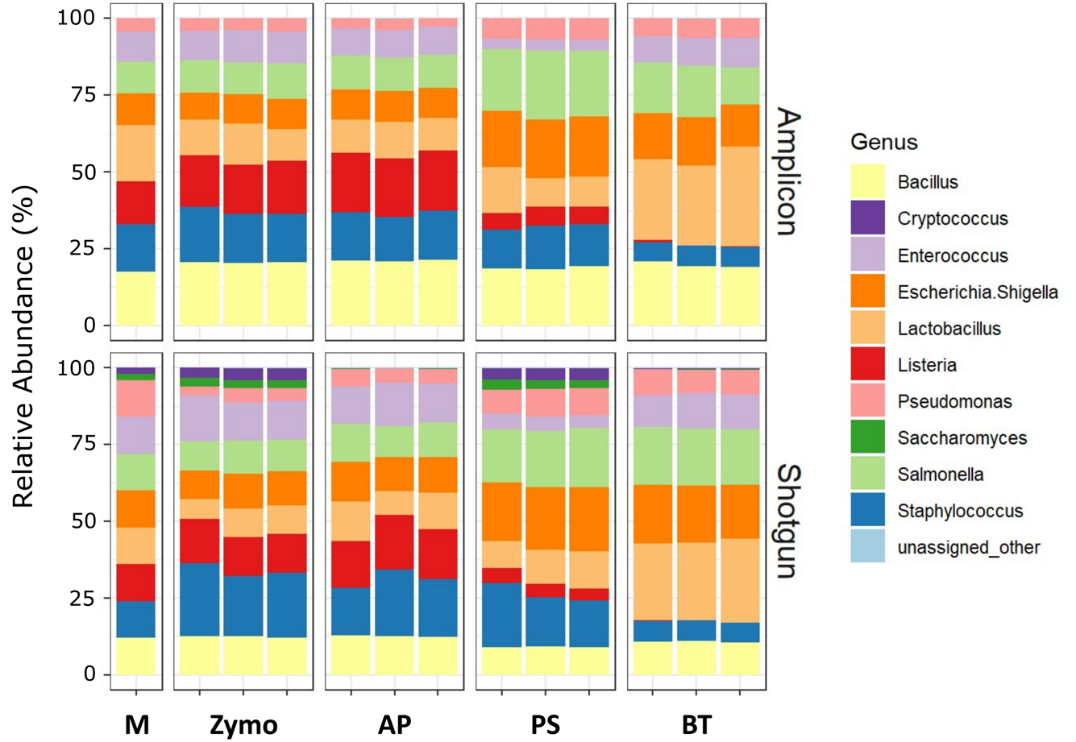
**

**B**

**
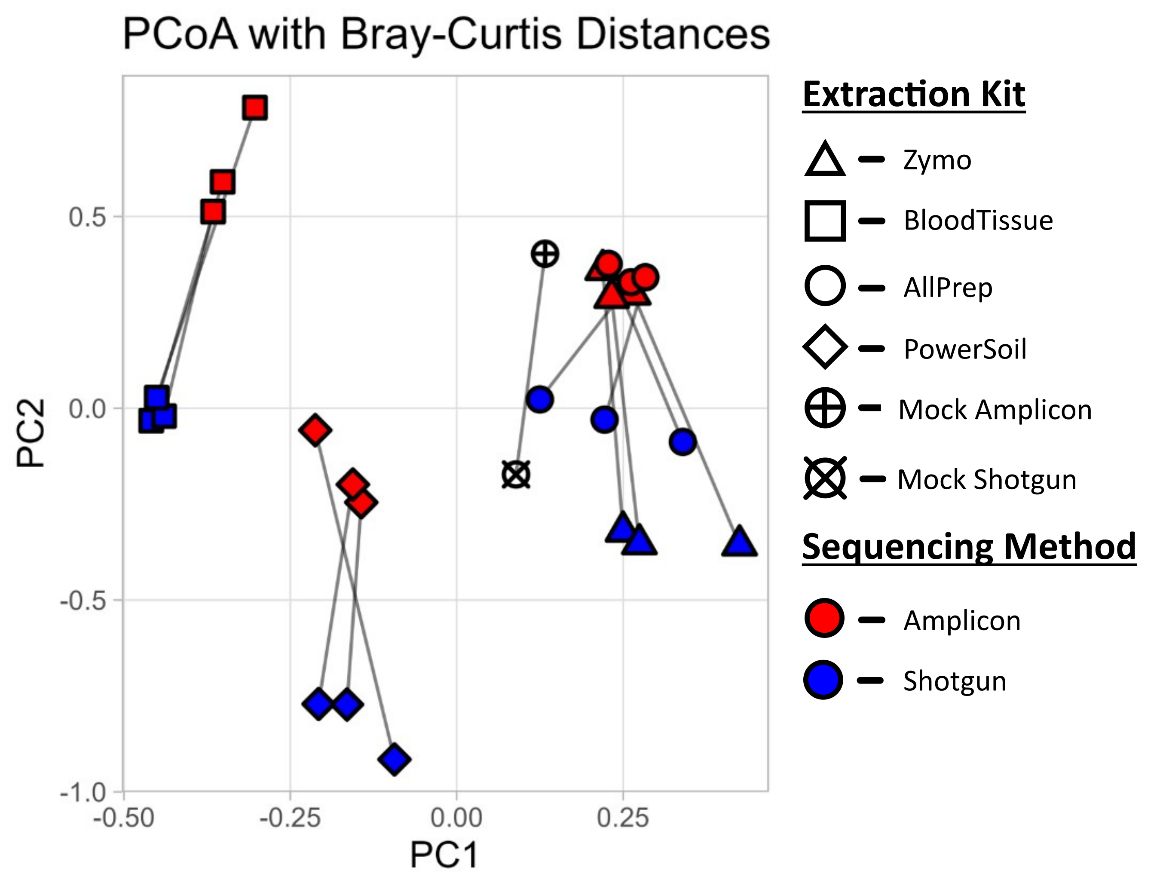
**

## **TABLE**

**Table 1**. Theoretical values and read characteristics of the mock microbial community analyzed by 16S rRNA amplicon sequencing. The values were raw relative abundance values from the amplicon sequence variants (ASV) table generated.

| Microorganism^a^ | 16S rRNA theoretical composition^b^ (%) | 16S rRNA copy number per genome | Read count per 16S rRNA sequence variant | Total # of reads |
| --- | --- | --- | --- | --- |
| *Pseudomonas aeruginosa* (B-3509) | 4.2 | 4 | 1.05×10^4^ | 4.2×10^4^ |
| *Escherichia coli* (B-1109) | 10.1 | 7 | 1.44×10^4^ | 1.01×10^5^ |
| *Salmonella enterica* (B-4212) | 10.4 | 7 | 1.49×10^4^ | 1.05×10^5^ |
| *Limosilactobacillus fermentum* (B-1840) | 18.4 | 5 | 3.68×10^4^ | 1.84×10^5^ |
| *Enterococcus faecalis* (B-537) | 9.9 | 4 | 2.48×10^4^ | 9.9×10^4^ |
| *Staphylococcus aureus* (B-41012) | 15.5 | 6 | 2.58×10^4^ | 1.55×10^5^ |
| *Listeria monocytogenes* (B-33116) | 14.1 | 6 | 2.35×10^4^ | 1.41×10^5^ |
| *Bacillus subtilis* (B-354) | 17.4 | 10 | 1.74×10^4^ | 1.74×10^5^ |
| Total | 100.0 | NA | NA | 1×10^6^ |

^a^Accession numbers are based on Agricultural Research Service Culture Collection (NRRL). The two fungal genomes are not shown as they are not picked up by 16S rRNA amplicon sequencing.

^b^The values for the microbial community standard lot used were provided by the manufacturer.
